# The context-dependent role of the Na^+^/Ca^2+^-exchanger (NCX) in pancreatic stellate cell migration

**DOI:** 10.1007/s00424-023-02847-3

**Published:** 2023-08-11

**Authors:** Thorsten Loeck, Micol Rugi, Luca Matteo Todesca, Paulina Kalinowska, Benjamin Soret, Ilka Neumann, Sandra Schimmelpfennig, Karolina Najder, Zoltán Pethő, Valerio Farfariello, Natalia Prevarskaya, Albrecht Schwab

**Affiliations:** 1 Institute of Physiology II, University of Münster, Robert-Koch-Straße 27b, 48149 Münster, Germany; 2 Université de Lille, Inserm, U1003 - PhyCell - Physiologie Cellulaire, F-59000 Lille, France; 3 Laboratory of Excellence, Ion Channels Science and Therapeutics, Villeneuve d’Ascq, France

**Keywords:** Na^+^/Ca^2+^-exchanger (NCX), Pancreatic stellate cells (PSCs), Migration, Ca^2+^, Tumor microenvironment (TME), Metastasis

## Abstract

Pancreatic stellate cells (PSCs) that can co-metastasize with cancer cells shape the tumor microenvironment (TME) in pancreatic ductal adenocarcinoma (PDAC) by producing an excessive amount of extracellular matrix. This leads to a TME characterized by increased tissue pressure, hypoxia, and acidity. Moreover, cells within the tumor secrete growth factors. The stimuli of the TME trigger Ca^2+^ signaling and cellular Na^+^ loading. The Na^+^/Ca^2+^ exchanger (NCX) connects the cellular Ca^2+^ and Na^+^ homeostasis. The NCX is an electrogenic transporter, which shuffles 1 Ca^2+^ against 3 Na^+^ ions over the plasma membrane in a forward or reverse mode. Here, we studied how the impact of NCX activity on PSC migration is modulated by cues from the TME. NCX expression was revealed with qPCR and Western blot. [Ca^2+^]_i_, [Na^+^]_i_, and the cell membrane potential were determined with the fluorescent indicators Fura-2, Asante NaTRIUM Green-2, and DiBAC_4_(3), respectively. PSC migration was quantified with live-cell imaging. To mimic the TME, PSCs were exposed to hypoxia, pressure, acidic pH (pH 6.6), and PDGF. NCX-dependent signaling was determined with Western blot analyses. PSCs express NCX1.3 and NCX1.9. [Ca^2+^]_i_, [Na^+^]_i_, and the cell membrane potential are 94.4 nmol/l, 7.4 mmol/l, and − 39.8 mV, respectively. Thus, NCX1 usually operates in the forward (Ca^2+^ export) mode. NCX1 plays a differential role in translating cues from the TME into an altered migratory behavior. When NCX1 is operating in the forward mode, its inhibition accelerates PSC migration. Thus, NCX1-mediated extrusion of Ca^2+^ contributes to a slow mode of migration of PSCs.

**Supplementary Information:**

The online version contains supplementary material available at 10.1007/s00424-023-02847-3.

## Introduction

Pancreatic ductal adenocarcinoma (PDAC) is the fourth leading cause of cancer-associated death in the USA [43]. It is projected to become the second leading cause of death in Germany in 2030 [32]. The high death rates are associated with the late onset of symptoms [36]. Fibrosis is a hallmark of PDAC. It is caused by the massive deposition of extracellular matrix that is produced by activated pancreatic stellate cells (PSCs) [7]. This phenomenon is called desmoplasia. Desmoplasia prevents efficient targeting of the tumor by the immune system and chemotherapeutic agents. PSCs are activated among others by tumor cell-derived growth factors such as PDGF, TGF-β, and TNF-α [14]. Matrix production changes the physico-chemical tissue properties in a way that leads to further activation of more PSCs. Eventually, tissue pressure rises to up to 100 mmHg which in turn leads to reduced O_2_ supply, resulting in hypoxic areas [31]. In addition, the modified metabolism of tumor cells creates an acidic environment [27, 30].

The modified tumor microenvironment (TME) triggers Ca^2+^ signaling through the activation of mechanosensitive ion channels and growth factors. Moreover, the activation of mechanosensitive ion channels as well as the necessity to defend the intracellular pH in the acidic TME in a Na^+^-dependent manner (e.g., by NHE1 or NBCe1 activation) leads to increased [Na^+^]_i_ loading [27]. Na^+^ loading is augmented because the Na^+^/K^+^-ATPase is inhibited in a hypoxic and acidic environment [35]. The Na^+^/Ca^2+^ exchanger (NCX) connects the Ca^2+^ and Na^+^ homeostasis. The NCX is electrogenic and transports 3 Na^+^ against 1 Ca^2+^ across the plasma membrane [3]. NCX removes Ca^2+^ from the cytosol in the forward mode, while NCX takes up Ca^2+^ in the reverse mode. The transport direction of NCX is determined by the Na^+^ and Ca^2+^ gradients and the membrane potential [3, 21].

Ca^2+^, in turn, is a crucial secondary messenger that plays a role in many cell functions such as proliferation or migration [5]. Migration is an important functional trait of PSCs since they can co-metastasize alongside tumor cells [48]. Migration critically depends on a fine-tuned regulation of the intracellular Ca^2+^ concentration ([Ca^2+^]_i_). It plays a role among others in the formation of new lamellipodia and the formation of new focal contacts [12, 45]. The signal transduction of specific signaling proteins affects migration. For instance, adhesion of cells is regulated by FAK. Moreover, well-known signaling pathways like AKT, MAPK, and YAP are known to have an impact on PSCs and Ca^2+^ signaling [23]. In addition, Ca^2+^ is required for the contraction of the cell body in the direction of migration [42] and localized cell shrinkage at the rear part of migrating cells [39, 41].

Here, we investigated the role of the NCX in the migration of PSCs with a particular focus on the conditions of the TME. In addition, signal transduction through the NCX was analyzed. For this purpose, signaling pathways such as Hippo, AKT, FAK, and MAPK signaling pathways were investigated, since these signaling pathways were shown to have an impact on migration [23].

## Methods

### Cells

We used primary murine pancreatic stellate cells and human stellate cells from the cell line PS1 in this study [8].

Murine PSCs were isolated from 8- to 12-week old male C57BL/6 wild-type mice. Experimental protocols were approved by the local committee for animal care (State Office for Nature, Environment and Consumer Protection of North Rhine-Westphalia; file number 81–02.05.50.20.003) and were performed according to current animal welfare guidelines.

The mice were sacrificed by cervical dislocation in isoflurane anesthesia. The pancreas was removed and washed with Grey’s balanced salt solution (GBSS). It was cut into little pieces, and the tissue was dissociated with collagenase P (2.5–3.0 mg, Roche) in 3 ml GBSS for 25 min. The resuspended cells were centrifuged and transferred to a FCS-coated tissue culture dish. After 2-h incubation at 37 °C with 5% CO_2_, the cells were washed 3–4 times to remove non-adherent cells. The remaining cells are PSCs which were cultured with DMEM-F12 with 10% FCS superior and 1% penicillin/streptomycin at 37 °C with 5% CO_2_. PSCs were cultured in medium at pH 7.4. The cells were used for the experiments in passage 2. We had shown previously that these culture conditions led to an activation of PSCs which we had determined by demonstrating a loss of VitA-containing NilRed-positive lipid droplets, an increase of αSMA expression and changes of morphology [33].

PS1 cells were cultured in DMEM-F12 with 10% FCS superior and 1% penicillin/streptomycin at 37 °C with 5% CO_2_.

### Quantitative real-time PCR

RNA was isolated from murine PSCs and human PS1 cells using Trizol (Gibco) followed by reverse transcription (Invitrogen). For cDNA synthesis, we used 1 µg of RNA for reverse transcription. The reaction mix contained: 10 µl Power Up SYBR Green master mix (Applied Biosystems), 1 µl forward primer and 1 µl reverse primer (each 10 µM), 6 µl H_2_O and 2 µl cDNA (20 ng), and 200 units superscript IV RT reverse transcriptase (Invitrogen). Quantitative real-time PCR was performed with a QuantStudio 3 (Applied Biosystems) thermal cycler. After the extension step of each cycle, the fluorescence intensity was measured. The relative changes in gene expression were calculated with the 2^−ΔCt^ method [26]. The qPCR protocol was the following: initial DNA denaturation (94 °C, 2 min) followed by 35 cycles of DNA denaturation (94 °C; 30 s), primer annealing (temperatures see Tables 1 and 2; 25 s), and DNA elongation (72 °C; 45 s). The primers were exon-exon overlapping and specific for the NCX splice variants. NCX expression was normalized to the geometric mean of GAPDH and YAWHAZ expression and GAPDH expression for murine samples and human samples, respectively. All experiments were performed in triplicates and repeated three times.

**Table 1:** Primer sequences for the murine NCX splice variants

Splice variants	Exons	Primer forward	Primer reverse	Melting temperature
NCX1.1	ACDEF	CCA GAA CGA TGA AAT AGT CAA AAC A	GAA GAT AGG TTG GCC ATA CAT C	60 °C
NCX1.2	BCD	CCA GAA CGA TGA AAT AGT GAA GAT C	AAT GTG AAG CCA CCA AGC TCA	59 °C
NCX1.3	BD	CCA GAA CGA TGA AAT AGT GAA GAT C	GTG AAG CCA CCT TTC AAT CCT	58 °C
NCX1.7	BDF	CCA GAA CGA TGA AAT AGT GAA GAT C	GAT AGG TTG GCC TGT TAA TGT G	60 °C
NCX1.9	BDE	CCA GAA CGA TGA AAT AGT GAA GAT C	CTT TTC CTG TTA ATG TGA AGC CAC	60 °C
NCX2	AC	TTG TGG AGA GCT GGA GTT CG	TTG GTT GAG TAG CAG AGC TGA G	58 °C
NCX3.1	AC	CAG TCAA AAC AAT TCA CAT CAA GG	GAG ATA ACA GGA GCG CTT TC	58 °C
NCX3.2	B	TGA AAC AGT GAA AAC CAT AAG GG	TCA CCC AAT ACT GGC TTT CCC	59 °C
NCX3.3	BC	GGA ATA TCA GCG CTC CTG TTA TC	TCA CCC AAT ACT GGC TTT CCC	59 °C
GAPDH	-	GAA GGT CGG TGT GAA CGG A	GAA GAT GGT GAT GGG CTT CC	59 °C
YWHAZ	-	GAA AAG TTC TTG ATC CCC AAT GC	TGT GAC TGG TCC ACA ATT CCT T	59 °C

**Table 2:** Primer sequences for the human NCX isoforms

Splice variants	Primer forward	Primer reverse	Melting temperature
NCX1	GGC CCT TAC CAT TAT CCG CA	TTG CTG TGC CAT CCT CTG TT	60 °C
NCX2	GCG ACG ACG AGA CCA TAG AA	CTG GCC CAG CTC AAT GAA GA	60 °C
NCX3	CAC CGT CAG TGC AGC AGG	CGG TGA GCA TGC CAA TGA TG	60 °C
GAPDH	AGG GGC CAT CCA CAG TCT TC	AGA AGG CTG GGG CTC ATT TG	60 °C

### Ionic imaging experiments

Ionic imaging experiments were performed for measuring the intracellular Ca^2+^ and Na^+^ concentrations ([Ca^2+^]_i_ and [Na^+^]_i_) and the membrane potential. Glass bottom dishes were coated with a 1:10 diluted collagen matrix. This matrix (1 ×) consists of 10.4 g/l RPMI, 10 mM HEPES, 40 µg/ml laminin, 40 µg/ml fibronectin, 800 µg/ml collagen I, 12 µg/ml collagen III, and 5.4 µg/ml collagen IV. The pH was set to 7.4 with NaOH [28]. The matrix polymerized overnight (14–18 h). PSCs were seeded the next day and cultured overnight. Before starting the measurements, bicarbonate-buffered medium was replaced by a HEPES-buffered medium, and the cells were allowed to adapt to the new medium for 1 h. The experimental setup consisted of an Axiovert 200 microscope (Zeiss), a sCMOS pco.edge camera (Excelitas PCO GmbH), and a VisiChrome polychromator (Visitron Systems GmbH). Data acquisition and analysis were controlled by VisiView software (Visitron Systems GmbH).

*[Ca*^*2*+^*]*_*i*_* measurements* were performed with the ratiometric Ca^2+^-indicator Fura-2-AM (3 µM; Invitrogen). The cells were stained at 37 °C for 30 min. After washing twice, the cells were superfused with Ringer’s solution (NaCl 140 mM, CaCl_2_ 1.2 mM, MgCl_2_ 0.8 mM, KCl 5.4 mM, HEPES 10 mM, glucose 5.5 mM, pH_e_ 7.4) at 37 °C. We employed a polychromator with a beam splitter 400DCLP and a D510/40m emission filter. The excitation wavelengths were 340 nm and 380 nm. The emission was measured at 510 nm. Images were taken in 10-s intervals. The fluorescence intensity was measured with VisiView software following background subtraction. For calibration, the cells were consecutively superfused with modified Ringer’s solutions with 0 Ca^2+^ (containing 5 mM EGTA) or 3 mM Ca^2+^. Both calibration solutions contained 2 µM ionomycin (Thermo Scientific). The [Ca^2+^]_i_ was calculated as described by Grynkiewicz [9]:

$${{[Ca}^{2+}]}_{i}={K}_{d}\times \beta \times \frac{\left(R-{R}_{\mathrm{min}}\right)}{\left({R}_{max}-R\right)}$$

where *K*_*d*_ is the dissociation constant of 224 nmol/l and *R*_*min*_ and *R*_*max*_ are the fluorescence ratios at zero and saturated Ca^2+^ after excitation with 340 and 380 nm. β is a factor of F_380_ with zero Ca^2+^ and F_380_ at saturated Ca^2+^ solutions.

*[Na*^+^*]*_*i*_* measurements* were done with the Na^+^ indicator Asante NaTRIUM Green-2 AM (ANG-2 AM, TEF Labs). The cells were stained with 5 µM ANG-2 at 37 °C for 1 h. After washing twice, the cells were superfused with Ringer’s solution at 37 °C. We used a beam splitter T525lpxr and an emission filter ET560/50 m. The excitation wavelength was 520 nm. Images were taken in 10-s intervals. For calibration modified Ringer’s solutions with 0, 10 and 20 mM Na^+^ were added to the cells. The Ringer’s solution with 0 Na^+^ contained N-methyl-D-glucamine (NMDG)-chloride 140 mM, HCl 122.5 mM, CaCl_2_ 1.2 mM, MgCl_2_ 0.8 mM, KCl 10 mM, HEPES 10 mM, and glucose 5.5 mM, pH_e_ 7.4. The 10 mM and 20 mM Na^+^ solution were produced by mixing the 0 Na^+^ solution with the respective amount of Ringer´s solution (composition see above). The calibration solutions contained 50 µM amphotericin B (Sigma). The mean fluorescence intensity of the projected cell area was determined after background subtraction. Following linear regression of the three calibration steps, the [Na^+^]_i_ concentration was calculated. Cells were only analyzed when the correlation factor of the linear regression was > 0.9.

*Membrane potential* was measured with the fluorescent dye DiBAC_4_(3) (bis-(1,3-dibutylbarbituric acid) trimethine oxonol; AAT Bioquest). Murine PSCs were stained with 2 µM DiBAC_4_(3) at 37 °C for 20 min. Two micromoles of DiBAC_4_(3) were also added to all solutions used during the course of the experiment. Cells were excited with a wavelength of 490 nm. We used an emission filter D535/25 m. For calibration, three modified Ringer’s solutions with 2 mM NaCl, 35 mM, and 140 mM NaCl were added to the cells; when necessary, NaCl was isosmotically replaced by NMDG. The 2 mM and 35 mM NaCl solution were produced by diluting the 0 Na^+^ solution (composition see above) with the respective amount of Ringer’s solution. Each calibration solution contained 1 µM gramicidin (Sigma-Aldrich). The Na^+^ concentrations of the calibration solutions were measured with a radiometer (ABL800 Flex, Radiometer). Mean fluorescence intensity of the projected cell area was determined after subtracting the background intensity. Following linear regression of the fluorescence intensities of the calibration steps, we used the Goldman-Hodgkin-Katz equation to calculate the membrane potential of PSCs. We assumed an intracellular K^+^ concentration of 155 mM and an intracellular Na^+^ concentration of 7 mM as determined during this study. Cells were only analyzed when the correlation factor of the linear regression of the calibration was > 0.9.

*NCX driving force*: The driving forces of the NCX are calculated using the following formula [1]:

$${\Delta G}_{NCX}=RT\times \mathrm{ln}{\left(\frac{{{[Na}^{+}]}_{e}}{{[Na}^{+}{]}_{i}}\right)}^{3}\times \frac{{{[Ca}^{2+}]}_{i}}{{[Ca}^{2+}{]}_{e}}-F\times {V}_{M}$$

Calculation of NCX driving forces requires the knowledge of intracellular and extracellular concentrations of Na^+^ and Ca^2+^, and the determination of the membrane potential (*V*_*M*_; *R* = 8.31 J mol^−1^K^−1^, *T* = 310.15 K, F = 96484.34 C mol^−1^).

### Mn^2+^ quench experiments

Ca^2+^ influx was assessed with the Mn^2+^ quench technique. PSCs were stained with 3 µM Fura-2-AM at 37 °C for 30 min. The cells were superfused with HEPES-buffered Ringer’s solution without Ca^2+^. Afterwards, the solution was changed to Ringer’s solution with or without Na^+^ containing 400 µM MnCl_2_ for 3–5 min. Mn^2+^ has a higher binding affinity to Fura-2-AM and quenches the fluorescence so that the rate of its decrease can be taken as a surrogate of Ca^2+^ influx. The microscope and filter settings were the same as for the [Ca^2+^]_i_ measurements. The excitation wavelength was 357 nm, and the emission was detected at 510 nm [22]. The experiments were performed at 37 °C. Images were acquired in 5-s intervals. We quantified the background-corrected cellular fluorescence intensity. Afterwards, the slope of the fluorescence decline was calculated for each time point of a 30-s period following the application of Mn^2+^ and plotted as relative quench rate (F/F_0_).

### siRNA transfection

A pool of four siRNA was used (GCAGACGCCUCCAUAGGUA, CCGAUUCUCUCUACUGUAA, GGAUUUCAUCUGUUAGUUA, GGAGAGACCACCAAGACAA; Dharmacon) to knock down expression of NCX1. Negative control siRNA (Qiagen) served as control. The transfections were performed with lipofectamine RNAiMAX (Invitrogen) according to the manufacturer’s protocol. In brief, 20,000 cells were seeded in a 3-cm glass bottom dish coated with a collagen III matrix. Nine microliters of lipofectamine were added in a tube with 150 µl Opti-MEM (Gibco). Three microliters of siRNA (10 µM) and 150 µl Opti-MEM (Gibco) were added to another tube. Both tubes were mixed 1:2 and incubated for 5 min at room temperature. The siRNA-lipid complexes were added to the cells. Fourty eight hours after transfection, the cells were used for migration and qPCR experiments.

### Cell migration experiments

Experiments were performed as 2D cell migration on a matrix (see ionic imaging section for its composition) onto which the cells were seeded. The matrix polymerized overnight (14–18 h). Afterwards, 25,000 murine or 20,000 human PSCs were seeded in T12.5 flasks or in 3-cm glass bottom dishes, respectively. The migration experiments were performed after overnight culturing. To mimic the TME conditions, the following conditions were tested: pH 6.6, 50 ng/ml PDGF, 100 mmHg pressure (above atmospheric pressure), and hypoxia (1% O_2_ or 0.5 mM DMOG). Hypoxia and DMOG were applied a few hours after seeding for 16–18 h. The other stimuli were applied 1 h before the migration experiment started. Migration of murine PSCs was recorded at 37 °C in CO_2_/HCO_3_^−^-buffered medium. When studying the effect of hypoxia and when recording migration of PS1 cells, we employed HEPES-buffered RPMI medium. The role of NCX1 was assessed either by its inhibition with 10 µM ORM-10103 in murine PSCs or by NCX1 knockdown in PS1 cells.

The cells were transferred into a pre-heated chamber (37 °C), and the migration was monitored with an inverted microscope (Axiovert 40C, Carls Zeis Inc.) connected to a camera (XC-77CE, Hamamatsu). The cell migration was recorded in 5-min intervals for 6 h. The time-lapse videos were analyzed with Amira® software, a self-made JAVA program and NIH ImageJ software. The parameters that describe the migration were derived from the cell trajectories that were reconstructed from the movement of the cell center. The distance between each time interval allows to calculate the speed (µm/min). It is determined as a three-point difference quotient. The translocation (µm) is the net distance covered during the course of the experiment.

### Western blots

Western blots were used to identify the NCX in PSCs and to analyze which signaling pathways are modulated by NCX blockade. For analysis of the signaling pathways, the murine PSCs were stimulated for 6 h with pH_e_ 7.4, pH_e_ 6.6, 50 ng/ml PDGF, or 100 mmHg pressure. 0.5 mM DMOG was added to the cells overnight (~ 16 h). Proteins were isolated with Pierce-RIPA-buffer (Thermo Fisher Scientific) containing 1 × Complete Mini (Roche) and 1 × PhosphoSTOP (Roche). The protein concentration was determined with the BCA protein assay kit (Thermo Fisher Scientific). Fifteen micrograms of protein were added in each lane of the 7.5% polyacrylamide-gels. Gels were run at 80 V. The protein transfer to a PVDF membrane was performed at 4 °C overnight. The membrane was blocked with 5% BSA in TBS-T (for GAPDH with 5% milk in TBS-T) for 1 h. Afterwards, the membrane was incubated with the first primary antibody at 4 °C overnight. The list of antibodies is shown in Table 3. After three times washing with TBS-T, an HRP-conjugated secondary antibody was added at room temperature for 1 h. After three times washing the detection was performed with a Clarity Max Western ECL Substrate (Bio-Rad) in the ChemiDoc™ XRS + Gel Imaging System (Bio-Rad). The quantification of the band intensities was done with Image Lab (Bio-Rad).

**Table 3:** Antibodies that were used to identify the NCX expression and the activity of different signaling pathways

Antibody	kDa	Phosphorylation site	Company	Secondary antibody
NCX (C2C12)	120 (fragments at 70 and 160 kDa)	-	Invitrogen (MA3-926)	Anti-mouse
FAK	125	-	Cell Signaling (3285S)	Anti-rabbit
p-FAK	125	Tyr397	Cell Signaling (3283S)	Anti-rabbit
YAP1	78	-	Santa Cruz Biotechnology (sc-101199)	Anti-mouse
p-YAP1	78	Ser127	Cell Signaling (4911S)	Anti-rabbit
AKT	60	-	Cell Signaling (9272)	Anti-rabbit
p-AKT	60	Ser473	Cell Signaling (9271S)	Anti-rabbit
p38 MAPK	42, 44	-	Cell Signaling (9212)	Anti-rabbit
p-p38 MAPK	42, 44	Thr180/Tyr182	Cell Signaling (9211S)	Anti-rabbit
GAPDH	38	-	Abcam (ab125247)	Anti-mouse

The antibodies were used in a concentration of 1:1000 (GAPDH in 1:5000) and were diluted in 5% BSA in TBS-T (GAPDH: 5% milk in TBS-T)

For analysis of the signaling proteins, the antibody detecting the phosphorylated signaling protein was used first, and then, the membrane was stripped to detect the total amount of the respective protein. GAPDH was used as a housekeeping protein. For stripping, the membrane was incubated in Aqua dest. for 5 min, followed by incubation in 0.2 M NaOH-solution for 5 min and finally again washing in Aqua dest. for 5 min. The membrane was blocked again for 1 h with 5% BSA in TBS-T.

### Statistical analysis

All experiments are shown as mean ± SEM. “N” stands for the number of repeated experiments with cells from different mice or different passages of the cell line PS1. “n” refers to the number of analyzed cells. All experiments were repeated at least three times. The statistical calculations were performed with “n”. The two-sided Student’s *t*-test was used for statistical analysis of two normally distributed and outlier-free groups. Outlier-free, non-normally distributed groups were analyzed with the Mann–Whitney *U* test. Experiments with more than two normally distributed groups were analyzed with one-way ANOVA test with Turkey’s multiple comparison test. Non-normally distributed groups were evaluated with the Kruskal–Wallis test with Dunn’s multiple comparison test. Multiple comparison tests over time were investigated with the repeated measure two-way ANOVA with Turkey’s multiple comparison test. For normalized data, the Wilcoxon signed-rank test was applied. Outliers were identified with the ROUT-outliers test (2%). A *p* value was defined as significant as *p* ≤ 0.05.

## Results

### Expression of NCX1 in pancreatic stellate cells

**Fig. 1:**
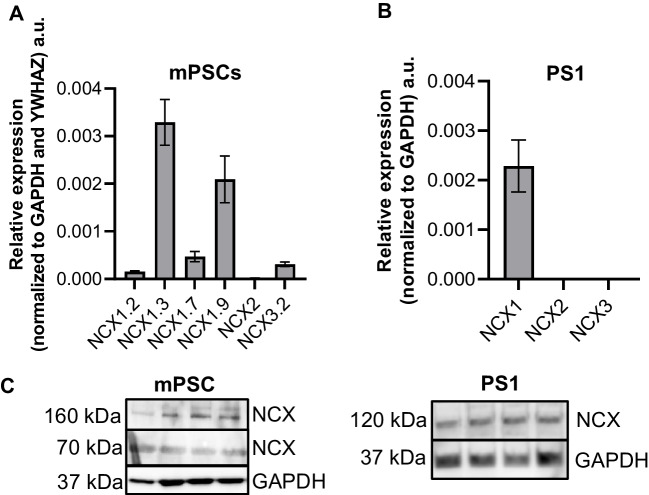
NCX1 isoform was expressed in murine and human PSCs. **A** NCX1.3 and NCX1.9 were the most frequently expressed NCX1 splice variants in murine PSCs (mPSC). **B** Human PS1 cells also expressed the NCX1 isoform. **C** Western blotting revealed the expression of the NCX1 in human (PS1) PSCs where we detected a band of the expected 120 kDa. In the murine protein samples, we detected bands at 70 kDa and 160 kDa which could represent a proteolytic fragment and the non-reduced exchanger, respectively [37]. **A** *N* = 4. **B** *N* = 3. **C** *N* = 4

The expression of the NCX in PSCs was investigated by quantitative real-time PCR and Western blot experiments. NCX1.3 and NCX1.9 were the most commonly expressed NCX splice variants in activated primary mouse PSCs (Fig. 1A). NCX1 is also the most abundant NCX isoform in the human stellate cell line PS1 (Fig. 1B). Western blot experiments confirmed the expression of NCX1 in PS1 cells and murine PSCs. In human PS1 cells, we detected NCX1 at the expected size of 120 kDa. In murine stellate cells, this band was not found. Instead, we detected bands at 70 kDa and 160 kDa that may represent a proteolytic fragment or an unreduced exchanger (Fig. 1C) [37].

**Fig. 2:**
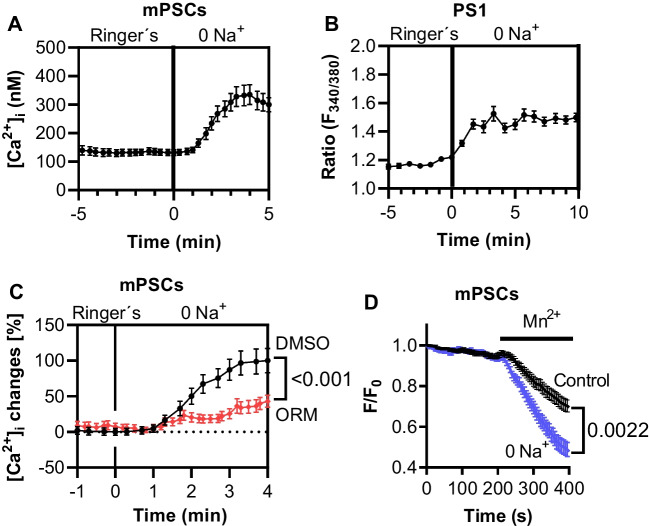
NCX1 was functionally expressed in PSCs. **A** Removal of extracellular Na^+^ (substituted by NMDG^+^) led to an increase of the [Ca^2+^]_i_ in murine PSCs (mPSCs) (*N* = 4; *n* = 34). **B** [Ca^2+^]_i_ rose in human PS1 after replacing Na^+^ in the superfusion solution by NMDG^+^(*N* = 4, *n* ≥ 146). **C** The application of the NCX inhibitor ORM-10103 attenuated the rise of the [Ca^2+^]_i_ upon extracellular Na^+^ removal (*N* = 4, *n* =  ≥ 29; repeated measure ANOVA). **D** Mn^2+^ quench experiments showed the increase of Ca^2+^ influx upon superfusing PSCs with 0 Na.^+^. These results indicate the reverse mode activity of the NCX1 (*N* = 4, *n* ≥ 39; repeated measure ANOVA)

Functional expression of NCX1 in murine PSCs is demonstrated by ion substitution experiments shown in Fig. 2. When extracellular Na^+^ was removed and replaced by NMDG, the cells responded with a rapid and reversible increase of the [Ca^2+^]_i_. The [Ca^2+^]_i_ values increased from 131.6 ± 10.2 nM with Ringer’s solution to 335.3 ± 34.8 nM with 0 Na^+^ solution (*N* = 4, *n* = 34; Fig. 2A). These findings are consistent with NCX operating in the reverse mode upon Na^+^ removal so that the [Ca^2+^]_i_ increased. We confirmed these results in the human PSC cell line PS1. The [Ca^2+^]_i_ also increased in PS1 cells when extracellular Na^+^ was replaced by NMDG^+^ (*N* = 4, *n* ≥ 146; Fig. 2B). Moreover, we applied the NCX inhibitor ORM-10103 when PSCs were superfused with Na^+^-free solutions. Then, the increase of the [Ca^2+^]_i_ was diminished. Since these experiments were performed in a paired fashion, we normalized the data to the respective control values (*N* = 4, *n* =  ≥ 29; Fig. 2C). Collectively, these results suggest that most of the [Ca^2+^]_i_ increase occured through the NCX. The conclusion that the NCX1 switched to the reverse mode upon Na^+^ removal was further supported by Mn^2+^ quenching measurements. The superfusion with 0 Na^+^ led to a higher Mn^2+^ influx compared to Ringer’s solution (Ringer’s: 0.14 ± 0.11 ΔF/F0/min vs. 0 Na^+^: 0.24 ± 0.02 ΔF/F0/min; *N* = 4, *n* ≥ 39; Fig. 2D). These results are consistent with the change of the transport mode to the reverse mode.

### NCX driving forces are pH-dependent

**Fig. 3:**
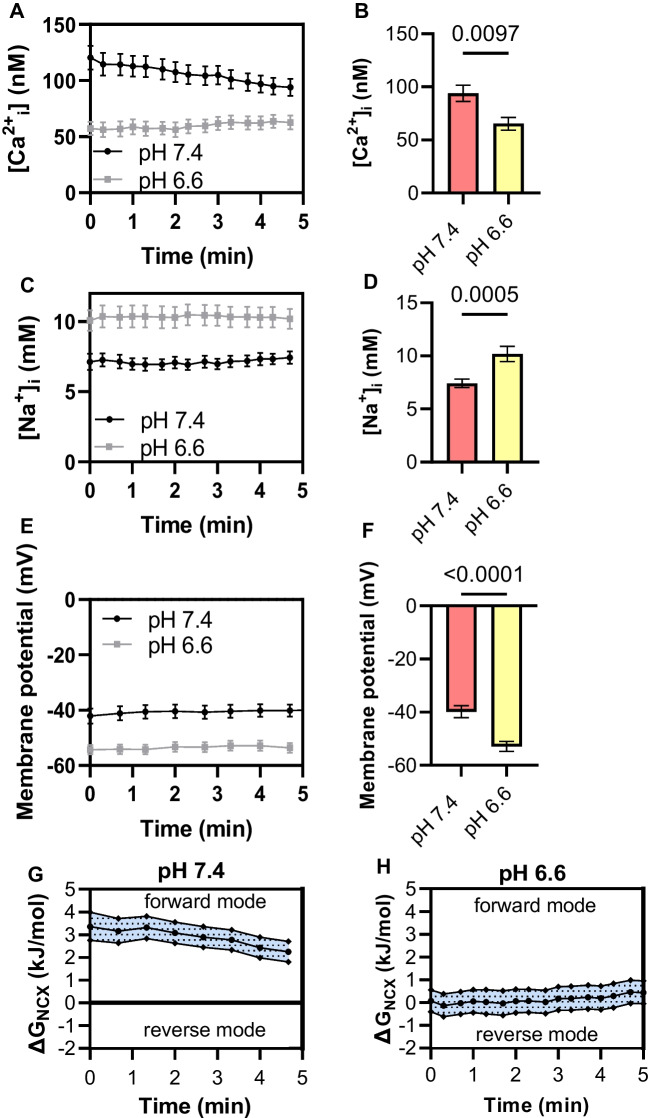
Determination of the driving forces of NCX1. **A**–**E** Measuring the [Ca^2+^]_i_ (**A**, **B**) and [Na.^+^]_i_ (**C**, **D**), the membrane potential (**E**, **F**) allowed to calculate the driving forces of the NCX (ΔG_NCX_; **G** + **H**). The measurements were done at pH_e_ 7.4 and pH_e_ 6.6. At pH_e_ 7.4, the driving forces predicted operation of the NCX1 in the forward mode (**G**). The driving forces were reduced in an acidic environment (**H**). **A**–**F** *N* = 4, *n* ≥ 40, Student’s *t*-test; **G**–**H** *N* = 4, *n* ≥ 30

Figure 3 illustrates ionic imaging experiments that were performed to determine the driving forces of NCX1 and to analyze whether they are pH-dependent. For this purpose, the [Na^+^]_i_, [Ca^2+^]_i_, and the membrane potential were measured while superfusing PSCs with physiological Ringer’s solution at pH_e_ 7.4 or at pH_e_ 6.6. From these data, the driving forces for the NCX could be calculated using the formula published by Baartscheer et al. [1] (see Methods). Under control conditions (pH_e_ 7.4), the [Ca^2+^]_i_ amounted to 94.4 ± 7.5 nM, and the [Na^+^]_i_ was 7.4 ± 0.4 mM (*N* = 4, *n* ≥ 40; Fig. 3A–D). The membrane potential was − 39.8 ± 2.2 mV (Fig. 3E + F). Given these values, the calculated driving forces ranged from 2.2 to 3.7 kJ/mol (Fig. 3G) indicating that NCX operated in the forward mode.

The driving forces were also determined when cells were exposed to an acidic extracellular pH of pH_e_ 6.6 in order to mimic the PDAC microenvironment [30]. Under these conditions, the [Ca^2+^]_i_ dropped to 65.3 ± 6.0 nM (Fig. 3A + B). In contrast, the [Na^+^]_i_ increased to 10.2 ± 0.7 mM, and the membrane potential hyperpolarized to − 52.9 ± 1.9 mV (*N* = 4, *n* ≥ 30; Fig. 3C–F). Based on these data, the mean driving forces of NCX ranged between 0.1 and 0.4 kJ/mol when PSCs were kept at pH_e_ 6.6 (*N* = 4, *n* ≥ 30; Fig. 3H). Thus, when PSCs are kept at pH_e_ 6.6, NCX1 is close to its reversal with only small driving forces for Ca^2+^ export.

### Inhibition of the NCX leads to a faster migration at pH_e_ 7.4

**Fig. 4:**
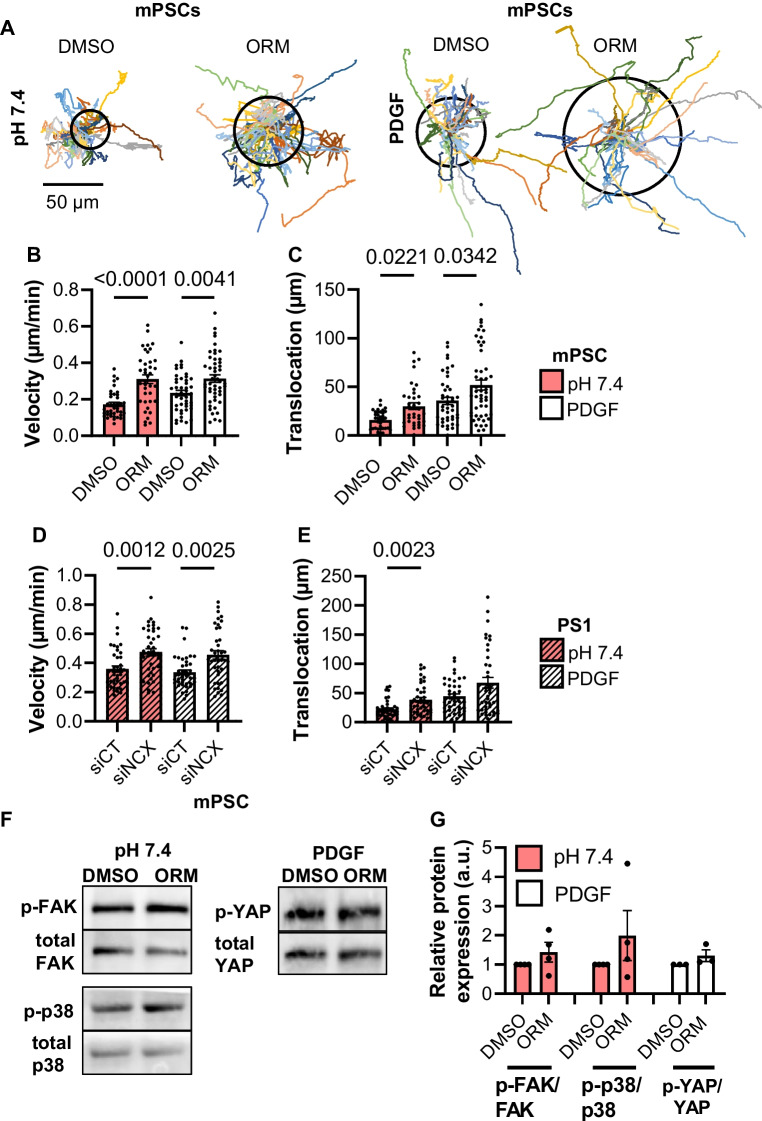
NCX1 inhibition stimulated migration of murine and human PSCs under control conditions and upon PDGF stimulation. **A** The trajectories of murine PSCs at pH_e_ 7.4 and stimulated with 50 ng/ml PDGF were normalized to common start points. The radii of the circles represent the mean translocation. **B** The velocity of murine PSCs (mPSCs) increased after NCX1 inhibition with 10 µM ORM-10103 (ORM) at pH_e_ 7.4. The same effect was detected after PDGF stimulation. **C** The translocation of murine PSCs increased after NCX1 inhibition with 10 µM ORM-10103 at pH_e_ 7.4 as well as after NCX1 blockade and PDGF stimulation. **D** The velocity of human PS1 cells increased after NCX1 knockdown (siNCX1) with siNCX1-RNA at pH_e_ 7.4 and after NCX1 knockdown and PDGF stimulation (siCT: control). **E** The translocation was increased after NCX1 knockdown at pH_e_ 7.4. PDGF-treated siNCX1-PS1 cells showed a tendency towards higher translocation. **A**–**E** *N* = 4, *n* ≥ 36. **B**–**E** Mann–Whitney *U* test. **F** + **G** NCX1 inhibition tended to increase phosphorylation of FAK (p-FAK) and p-38-MAPK (p-p38) at pH_e_ 7.4 as well as that of YAP (p-YAP) after PDGF stimulation. *N* ≥ 3, Wilcoxon signed-rank test

To analyze the migratory behavior of murine and human PSCs at pH_e_ 7.4, spontaneous undirected migratory movements were recorded by live-cell imaging for 6 h. These experiments are depicted in Fig. 4. NCX1 was either inhibited in primary murine PSCs with 10 µM ORM-10103 [17], or NCX1 was silenced using siRNA in the human PSC cell line PS1. With this approach, it was possible to verify the results obtained in murine PSCs with a knockdown method.

Both maneuvers, pharmacological blockade or NCX1 silencing, produced comparable effects. Migration of murine and human stellate cells was stimulated so that velocity and translocation increased (velocity: DMSO: 0.18 ± 0.01 µm/min vs. ORM-10103: 0.31 ± 0.02 µm/min; siCT: 0.36 ± 0.02 µm/min vs. siNCX1: 0.48 ± 0.03 µm/min; translocation: DMSO 15.9 ± 1.6 vs. ORM-10103 30.1 ± 3.5; siCT: 22.2 ± 2.5 vs. siNCX1: 38.5 ± 4.1; *N* = 4, *n* ≥ 35; Fig. 4A–E). These data suggest that an active NCX1 slows down the migration of PSCs.

### NCX1 inhibition accelerates migration of PDGF-stimulated murine and human PSCs

Tumor cells secrete various growth factors that stimulate and activate PSCs [27]. Here, we tested whether the stimulation of stellate cell migration with PDGF is regulated by NCX1 when cells are kept at pH_e_ 7.4 (see Fig. 4). The application of PDGF led to a faster migration of stellate cells as previously shown [46]. Migration of murine and human PSCs was further accelerated upon NCX1 inhibition and silencing, respectively (murine PSCs: DMSO: 0.23 ± 0.02 µm/min; ORM-10103: 0.31 ± 0.02 µm/min; human PSCs: siCT: 0.33 ± 0.02 µm/min; siNCX1: 0.46 ± 0.03 µm/min; *N* = 4, *n* ≥ 35; Fig. 4A–E). The translocation of murine PSCs increased after NCX blockade (DMSO: 35.7 ± 3.7 µm; ORM-10103: 51.8 ± 5.1 µm; *N* = 4, *n* ≥ 35; Fig. 4C). As far as signaling pathways are concerned, we found a trend towards more phosphorylation of YAP upon NCX1 inhibition in PDGF-stimulated murine PSCs (Fig. 4F + G).

### Inhibition of the NCX1 leads to a slower migration at pH_e_ 6.6

**Fig. 5:**
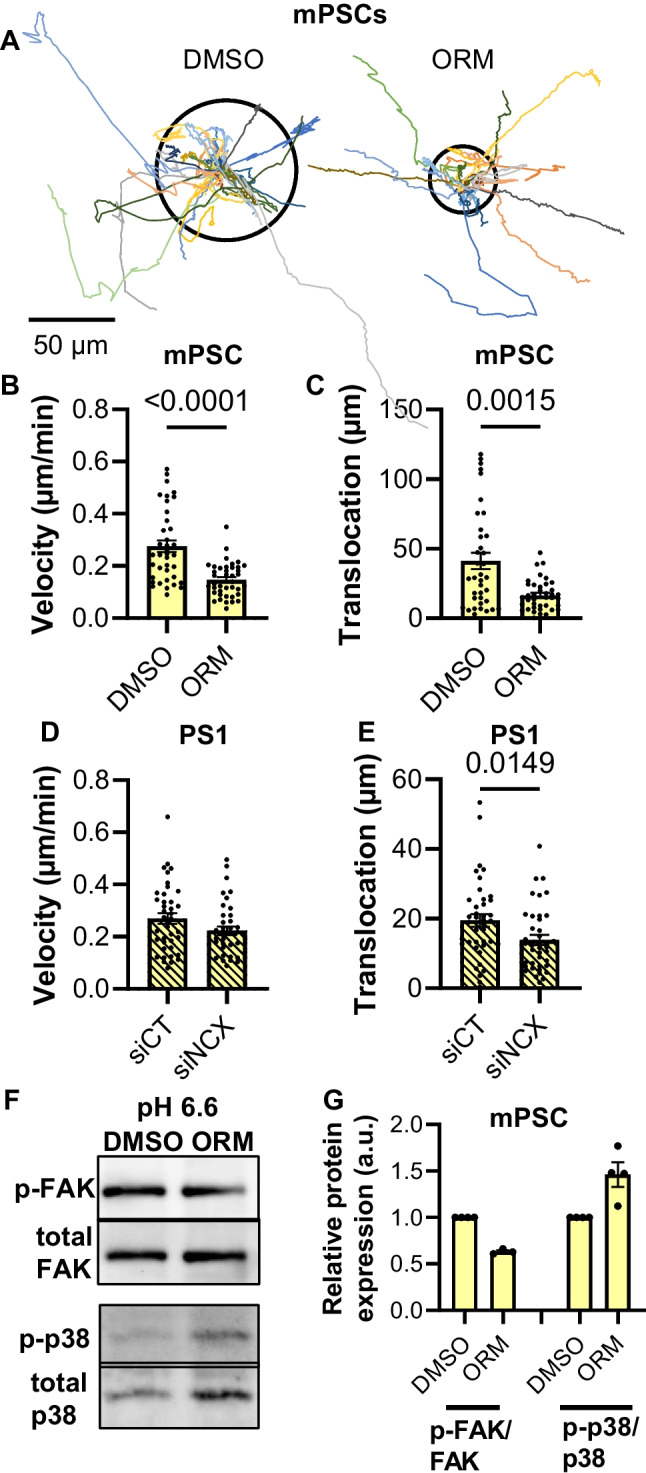
NCX inhibition in an acidic environment (pH_e_ 6.6) led to a reduced migration. **A** Trajectories of PSCs treated with pH_e_ 6.6 normalized to common starting points. The radii of the circles represent the mean translocation. **B** The velocity of murine PSCs (mPSCs) was reduced after NCX1 inhibition with ORM-10103 (ORM). **C** The translocation of murine PSCs was lower after NCX1 inhibition. **D** The velocity of human PS1 was not different after NCX1 inhibition (siNCX1; control: siCT). **E** The translocation was reduced after NCX1 knockdown in acidic pH_e_. *N* = 4, *n* ≥ 37, Mann–Whitney *U* test. **F** + **G** FAK presented a tendency to be less activated when the NCX1 was inhibited, while p-38 displayed a higher activation when the NCX1 was blocked in acidic pH_e_. *N* ≥ 3, Wilcoxon signed-rank test

Migration of PSCs was also tested at an acidic extracellular pH_e_ 6.6 (see Fig. 5) which is a characteristic feature of the PDAC microenvironment. Under these conditions, murine PSCs migrated faster than at pH_e_ 7.4 (*p* = 0.0002). However, inhibition of the NCX caused a significant decrease of the velocity of murine PSCs. We observed a similar tendency in human siNCX1-PSCs (murine PSCs: DMSO: 0.27 ± 0.02 µm/min; ORM-10103: 0.15 ± 0.01 µm/min; human PSCs: siCT: 0.27 ± 0.02 µm/min; siNCX1: 0.22 ± 0.1 µm/min; *N* = 4, *n* ≥ 38; Fig. 5B + D). We found the same effects for the translocation (murine PSCs: DMSO: 44.0 ± 6.3 µm vs. ORM-10103: 17.7 ± 2.1 µm; human PSCs: siCT: 19.5 ± 1.8 vs. siNCX1: 13.9 ± 1.5 µm; *N* = 4, *n* ≥ 38, Fig. 5A, C + E).

We analyzed FAK phosphorylation in murine PSCs. Although the changes did not reach statistical significance, it was noteworthy that there were opposing trends in FAK phosphorylation when NCX1 was inhibited at pH_e_ 7.4 and at pH_e_ 6.6 (Figs. 4G and 5G). The trends in FAK phosphorylation mimicked the pH-dependent changes of cell migration caused by NCX1 inhibition.

### Inhibition of NCX1 upon pressure application results in altered migration behavior in murine and human PSCs

**Fig. 6:**
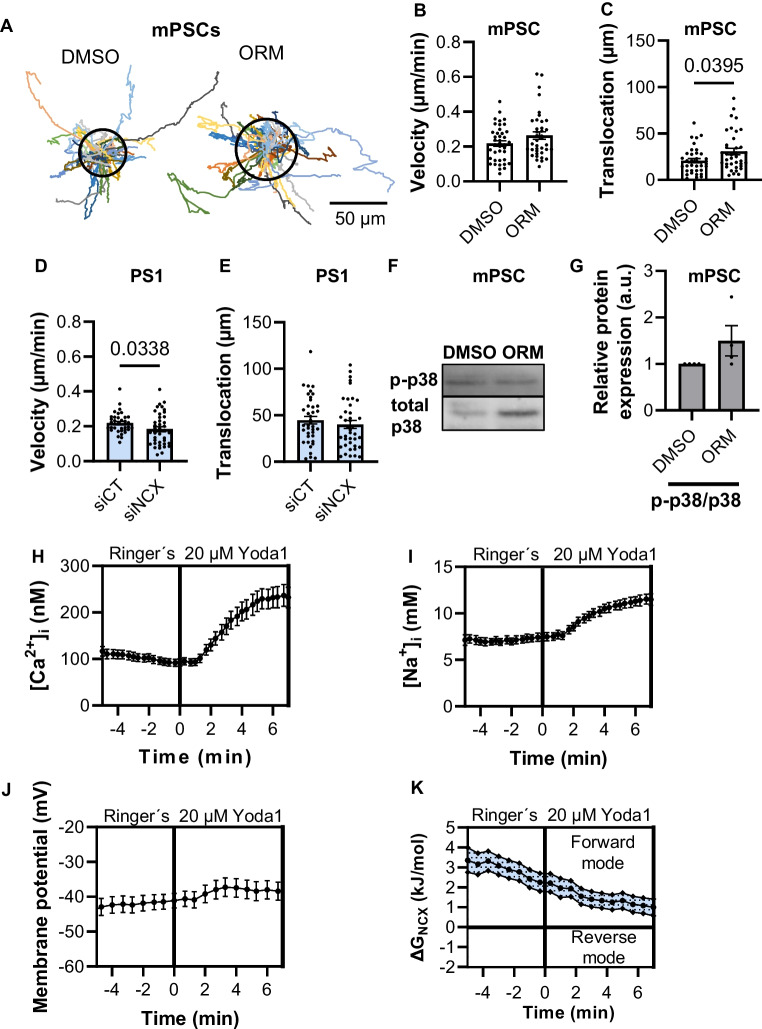
The application of pressure (100 mmHg) led to differential responses of murine and human PSCs upon NCX1 inhibition or knockdown. **A** The trajectories (normalized to common starting points) of murine PSCs (mPSCs) with 100 mmHg pressure application. The radii of the circles represent the mean translocation. **B** In murine PSCs, the velocity was not changed upon NCX1 inhibition (ORM-10103). **C** The translocation increased after NCX1 inhibition with 10 µM ORM-10103 (ORM). **D** The velocity decreased of siNCX1-PS1 cells. **E** The translocation of siNCX1-PS1 cells was not changed. **B**–**E** *N* = 4, *n* ≥ 37, Mann–Whitney *U* test. **F** + **G** A tendency towards higher p-38 phosphorylation was shown under pressure treatment with NCX inhibition. *N* ≥ 3, Wilcoxon signed-rank test. **H**–**K** Piezo1 activation led to a reduction of the NCX driving forces. Murine PSCs were analyzed for the concentration of [Ca^2+^]_i_ (**H**), [Na.^+^]_i_ (**I**), and the membrane potential (**J**) to calculate the driving forces of the NCX (**K**). **F**–**I** *N* ≥ 4, *n* ≥ 43

Fibrosis in PDAC is accompanied by a massively elevated tissue pressure by up to 100 mmHg [31]. To mimic this property of the PDAC microenvironment, we placed PSCs into a pressure chamber that maintained a pressure of 100 mmHg above the ambient pressure. Murine and human PSCs responded differentially to NCX1 inhibition when they were exposed to elevated pressure as shown in Fig. 6. Translocation of murine PSCs was increased upon NCX1 inhibition (DMSO: 20.8 ± 2.8 µm; ORM-10103: 30.7 ± 3.5 µm; *N* = 4, *n* ≥ 38; Fig. 6A–C). However, a decrease in velocity was observed in human PSCs (siCT: 0.22 ± 0.01 µm/min; siNCX1: 0.18 ± 0.01 µm/min; *N* = 4, *n* ≥ 37; Fig. 6D + E). There was a trend towards stronger phosphorylation of p38 when NCX1 was inhibited in pressurized PSCs (Fig. 6F + G).

Pressure leads to the activation of mechanosensitive ion channels such as Piezo1 and K_2P_2.1 (TREK1) [29]. Opening of these channels can be expected to change NCX1 driving forces and hence NCX1 function [1, 29]. We showed this exemplarily by determining the [Ca^+2+^]_i_, [Na^+^]_i_, and the cell membrane potential of PSCs following activation of Piezo1 channels with 20 µM Yoda1 in Ringer’s solution. The [Ca^+2+^]_i_ and [Na^+^]_i_ rose to 232.6 ± 21.9 nM and 11.5 ± 0.5 mM (*N* = 4, *n* ≥ 40; Fig. 6H + I), respectively, and the membrane potential depolarized to − 38.4 ± 3.1 mV (*N* = 4, *n* = 39; Fig. 6J). Collectively, this led to a decrease of the driving force in the forward mode (Fig. 6K).

### NCX1 inhibition does not affect migration of PSCs kept in hypoxia

**Fig. 7:**
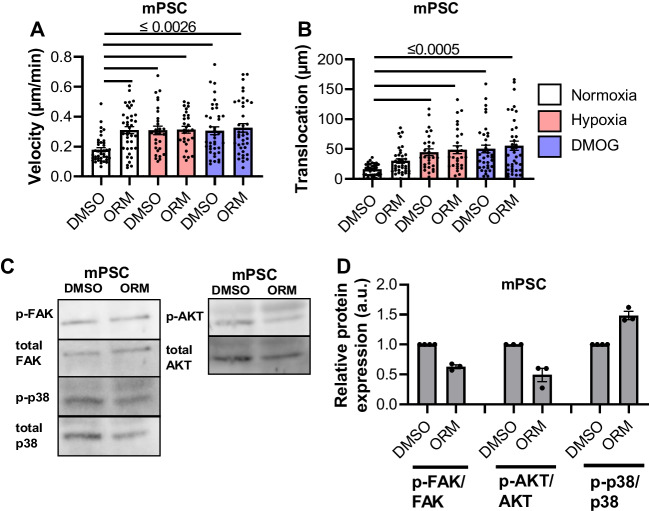
Hypoxia led to a faster migration of murine PSCs. **A** + **B** The velocity (**A**) and translocation (**B**) of murine PSCs increased in hypoxia (1% O_2_ and 0,5 mM DMOG). No NCX1-related effects were observed (DMSO: control, ORM: NCX1 inhibition). **C** + **D** There was a trend towards reduced phosphorylation of FAK and AKT and increased phosphorylation of p-38-MAPK upon NCX1 inhibition in PSCs exposed to hypoxia. **A** + **B** *N* = 4, *n* =  ≥ 36, Kruskal–Wallis test with Dunn’s multiple comparison test. **C** + **D** *N* = 3, Wilcoxon signed-rank test

In the tumor microenvironment, the increased tissue pressure causes the vessels to collapse. As a result, less O_2_ reaches the cells, and they have to adjust their metabolism [25]. In the migration experiments shown in Fig. 7, hypoxia was applied either by culturing the cells in 1% O_2_ or by administering dimethyloxalylglycine (DMOG). DMOG prevents the degradation of HIF-1α and thereby induces chemical hypoxia [15]. As shown previously, hypoxia accelerated PSC migration [28]. However, the additional inhibition of NCX1 did not cause any further modulation of the migratory behavior (DMSO 0.18 ± 0.01 µm/min; hypoxia DMSO 0.31 ± 0.03 µm/min; hypoxia ORM-10103 0.31 ± 0.02 µm/min; *N* = 4, *n* =  ≥ 36; Fig. 7A + B).

The analysis of signaling proteins revealed opposing tendencies of phosphorylation. Phosphorylation of FAK and AKT was reduced upon NCX1 inhibition while that of p38-MAPK increased upon NCX inhibition (Fig. 7C + D).

### The combined application of microenvironmental cues abrogates the impact of NCX1 inhibition on PSC migration

**Fig. 8:**
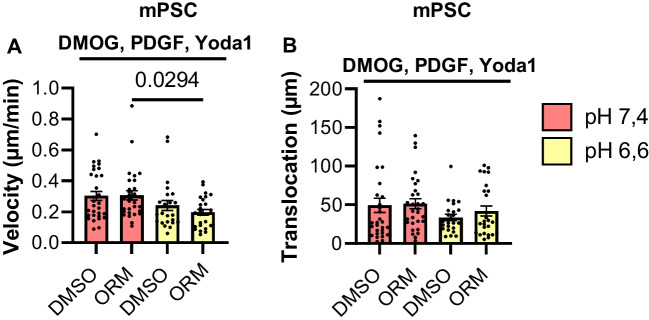
Combining multiple cues from the microenvironment rendered PSCs insensitive to NCX1 inhibition. **A** + **B** The velocity (**A**) and translocation (**B**) did not differ between the control treatment (DMSO) and NCX1 inhibition (ORM) at pH_e_ 7.4 and pH_e_ 6.6. The murine PSCs were treated with 0.5 mM DMOG, 50 ng/ml PDGF, und 20 μM Yoda1 to mimic the combination effects of the tumor microenvironment. *N* ≥ 3; *n* ≥ 30, Kruskal–Wallis Test with Dunn’s multiple comparison test

Finally, we treated murine PSCs with a combination of DMOG to mimic hypoxia, 50 ng/ml PDGF, and 20 µM Yoda1 to activate the mechanosensitive ion channels Piezo1. These experiments, shown in Fig. 8, were performed at pH_e_ 7.4 and pH_e_ 6.6. Compared with control data at pH_e_ 7.4 (Fig. 4), faster migration was observed upon stimulation with DMOG, PDGF and Yoda1 (pH_e_ 7.4, DMSO: 0.18 ± 0.01 µM/min, Fig. 4B; pH_e_ 7.4, DMOG, PDGF, Yoda1, DMSO: 0.24 ± 0.03 µm/min; *p* = 0.0002; Mann–Whitney *U* test; *N* ≥ 3, *n* ≥ 30; Fig. 8A + B). It appears that hypoxia had the strongest effect on stellate cell migration. This could explain the higher velocity. Moreover, the effect of the acidosis seems to be lower in the combination treatment. However, the effect of NCX1 on migration was not observed in the combination treatment as we did not observe any effect of NCX1-inhibition with ORM-10103.

## Discussion

It is well established that ion channels and transporters contribute to cancer progression. Yet, a more detailed understanding of their functional role in tumor and, in particular, in stromal cells is still needed for evaluating the therapeutic potential of targeting transport proteins. Pancreatic stellate cells play a crucial role in the progression of PDAC [7]. By switching from the quiescent to the activated state which results in a greatly increased production of ECM proteins, PSCs contribute to disease progression [47]. Numerous studies have shown that PSC functions depend on a fine-tuned regulation of their [Ca^2+^]_i_ and thereby on the activity of Ca^2+^ transport proteins [7, 10, 11, 28, 34, 46]. Here, we identified another piece of the “Ca^2+^ toolkit” of PSCs, the Na^+^/Ca^+^ exchanger NCX1. It provides as direct link between Ca^2+^ signaling and the cellular Na^+^ homeostasis which is stressed by the properties of the TME. So far, the function of the NCX in cancer-associated fibroblasts is not well understood [27]. Therefore, we undertook a systematic characterization of the NCX1 in PSCs.

Performing quantitative real-time PCR and Western blot experiments, we detected distinct NCX1 splice variants in pancreatic stellate cells. The NCX1 isoform is common in most tissues [21]. The knowledge of the splice variants is relevant since they have characteristic pharmacological properties [13]. The interpretation of Western blot experiments was not unambiguous because we did not detect the murine NCX1 at the expected size of 120 kDa. The 70 kDa and 160 kDa bands found in our experiments have previously been described as a proteolytic fragment and a reduced form of the exchanger, respectively [37]. However, by showing an ORM-10103–dependent decrease of the [Ca^2+^]_i_ in Na^+^-free Ringer’s solution, we could provide strong functional evidence for NCX1 activity in PSCs. The rise of the [Ca^2+^]_i_ upon removing Na^+^ indicates the change of NCX1 to reverse mode (Ca^2+^ influx) [6].

After showing the functional expression of NCX1 in PSCs, we determined its driving forces at different pH_e_ values and following the activation of the mechanosensitive channel Piezo1 by measuring the intracellular Ca^2+^ and Na^+^ concentrations as well as the membrane potential. These indicated NCX1 activity in the forward mode at pH_e_ 7.4, which is the case for most cells [3, 20]. Due to the altered energy production by lactate and glycolysis of the tumor cells, many protons are generated in the tumor. The acidic pH_e_ of the TME will also impact on PSCs. It will induce Na^+^ loading because of increased Na^+^-dependent H^+^ export when the Na^+^/K^+^-ATPase is partially inhibited [35]. Consequently, NCX1 driving forces are strongly reduced at pH_e_ 6.6. In addition, NCX1 itself is inhibited by protonation so that NCX1 activity will be low in an acidic environment [16].

PSCs can co-metastasize with tumor cells [48]. Ca^2+^ is a secondary messenger that is crucial for cell migration by regulating among others the formation of lamellipodia, retraction of the cell body, and organization of the cytoskeleton [40, 42]. The role of NCX in cell migration is discussed controversially. Some studies came to the conclusion that the NCX suppresses migration [19, 38]. In contrast, another study showed increased cell migration with decreased NCX expression [2]. Our results clearly showed that the influence of NCX1 on migration was strongly context-dependent. The properties of the microenvironment dictated whether NCX1 dampened or enhanced migratory activity. Conversely, depending on the microenvironment, NCX1 blockade resulted either in a stimulation or in an inhibition of PSC migration.

At pH_e_ 7.4, faster migration was observed upon NCX1 inhibition. NCX1 operated effectively in the forward mode under these conditions so that [Ca^2+^]_i_ was removed from the cell. If this was no longer possible upon NCX1 inhibition, an increase of the [Ca^2+^]_i_ would occur. A moderate increase of the [Ca^2+^]_i_ led to faster migration [28]. A similar effect was observed when NCX1 was inhibited in PDGF-stimulated PSCs. Although we did not determine NCX driving forces under these conditions, NCX1 was expected to work in the forward mode as well. We had shown earlier that PDGF led to a (transient) elevation of the [Ca^2+^]_i_ and a concomitant activation of K_Ca_3.1 channels [46]. Both would favor the forward mode of NCX1. Hence, NCX1 inhibition elicited similar effects as under control conditions.

At first sight, it was surprising that NCX1 inhibition did not affect PSC migration when they were kept under hypoxia. However, it was conspicuous that PSCs migrated faster and farther than under any other condition. This result was consistent with our previous results [28] and could at least be partially explained by increased cytokine release. Yet, the question remained why NCX1 had no further effect. One explanation is that PSCs were already maximally stimulated and thereby migrating at their maximal velocity so that any NCX1-dependent modulation was overridden. Moreover, NCX1 activity was already quite low because hypoxia led to an inhibition of the Na^+^/K^+^-ATPase [4] and consequently to less favorable gradients for NCX1 forward mode.

At pH_e_ 6.6, the opposite behavior was observed with NCX1 inhibition causing an impairment of PSC migration. Here, the acid might act on additional ion channels, transporters, and receptors as well as on the cell surface pH [24, 44] that had not been investigated in this study. In the literature, it was described that cells needed an optimal (cell surface) pH value for migration. In melanoma cells, the velocity and translocation were decreased at pH_e_ 6.4 [44], while it increased in breast cancer cells when NHE1 was inhibited [24]. It was also shown in melanoma cells that signaling of the G-protein coupled H^+^ receptor GPR4 linked the activity of FAK to the ambient pH [18]. FAK plays a role in the formation of integrins for cell adhesion, and increased FAK activity was associated with increased migration [49]. Our results do not allow to conclude with certainty that the opposing effects of NCX1 inhibition at pH_e_ 7.4 and pH_e_ 6.6 on migration were related to FAK phosphorylation. Nonetheless, we note that there is a trend towards higher FAK activity upon NCX1 inhibition at pH_e_ 7.4 and lower at pH_e_ 6.6. Thus, NCX1-induced changes of FAK phosphorylation may mimic those of PSC migration. Moreover, a further increase of the [Ca^2+^]_i_ upon NCX1 inhibition potentially constituted too much stress for PSCs so that their migration decreased. We had seen a comparable cytoskeleton-dependent behavior earlier. Piezo1 activation elicited increased force generation by PSCs at pH 7.4, while force generation decreased when Piezo1 was activated in an acidic environment [22].

When pressure was applied to the cells, different responses were observed in murine and human cells. Pressure likely activated mechanosensitive ion channels such as Piezo1 and thereby led to an increase of the [Ca^2+^]_i_ and of the [Na^+^]_i_ as we showed in our experiments. Accordingly, driving forces for NCX1 forward mode could be expected to decrease following the activation of mechanosensitive ion channels such as Piezo1. Thus, NCX1 activity was possibly too low to achieve a strong effect on migration.

In the TME, the different environmental cues usually occur in combination [27]. We mimicked this situation in our last set of experiments by combining all of the above tested stimuli. We detected no NCX1-dependent modulation of the migration under these conditions. However, we noted that migration was as fast as it was under hypoxic conditions. It therefore appeared as if hypoxia was the strongest microenvironmental stimulus that was dominant over the other stimuli. Migration was as fast as in hypoxia alone, and it could not be modulated by NCX1 inhibition.

**Fig. 9:**
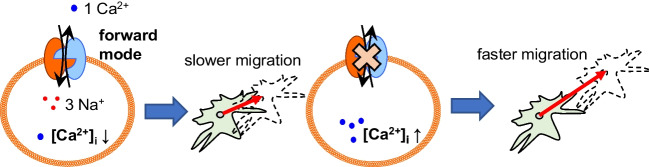
Effects of NCX1 on PSC migration. Forward mode activity of NCX1 led to a slower migration. Pharmacological inhibition of NCX1, downregulation with siRNA or low NCX1 activity resulted in faster migration. The migration of PSCs depends on how the prevailing environmental conditions modulate the NCX1

In summary, this work provided evidence for the functional expression of NCX1 in murine and human stellate cells. It was also shown that NCX1 modulated the migration of PSCs in a way that depended on the prevailing environmental conditions (Fig. 9). Thus, NCX1 inhibition may be more likely to contribute to metastasis in cells located at the periphery of the tumor, where pH_e_ is less acidic, and pressure is lower. An active NCX1 in the tumor periphery operating in the forward mode would be more likely to prevent metastasis. When NCX1 is active in the forward mode, it contributes to the slowing down of the cells by removing [Ca^2+^]_i_ from the cell. When NCX1 is inhibited or inactive, the [Ca^2+^]_i_ rises so that faster and further migration occurs.

### Supplementary Information

Below is the link to the electronic supplementary material.

Supplementary file1 (PPTX 991 KB)

## Data Availability

Not applicable.
