# Correction to: The context‑dependent role of the Na^+^/Ca^2+^‑exchanger (NCX) in pancreatic stellate cell migration

**DOI:** 10.1007/s00424-023-02874-0

**Published:** 2023-11-03

**Authors:** Thorsten Loeck, Micol Rugi, Luca Matteo Todesca, Paulina Kalinowska, Benjamin Soret, Ilka Neumann, Sandra Schimmelpfennig, Karolina Najder, Zoltán Pethő, Valerio Farfariello, Natalia Prevarskaya, Albrecht Schwab

**Affiliations:** 1 Institute of Physiology II, University of Münster, Robert‑Koch‑Straße 27b, 48149 Münster, Germany; 2 Université de Lille, Inserm, U1003 - PhyCell – Physiologie Cellulaire, F‑59000 Lille, France; 3 Laboratory of Excellence, Ion Channels Science and Therapeutics, Villeneuve d’Ascq, France

**Correction to: Pflügers Archiv - European Journal of Physiology (2023) 475:1225–1240**

10.1007/s00424-023-02847-3

The originally published article contains an error. One author was mistakenly not listed and this should be corrected (Paulina Kalinowska).

The original article has been corrected.

