# Supplementary material for: The context-dependent role of the Na+/Ca2+-exchanger (NCX) in pancreatic stellate cell migration
Source: Pflugers Arch. 2023 Aug 11;475(10):1225–40. doi: 10.1007/s00424-023-02847-3 (PMC10499968; doi:10.1007/s00424-023-02847-3)
Supplement: Supplementary file 1 — Supplementary file1 (PPTX 991 KB) [file 424_2023_2847_MOESM1_ESM.pptx]

## Slide 1
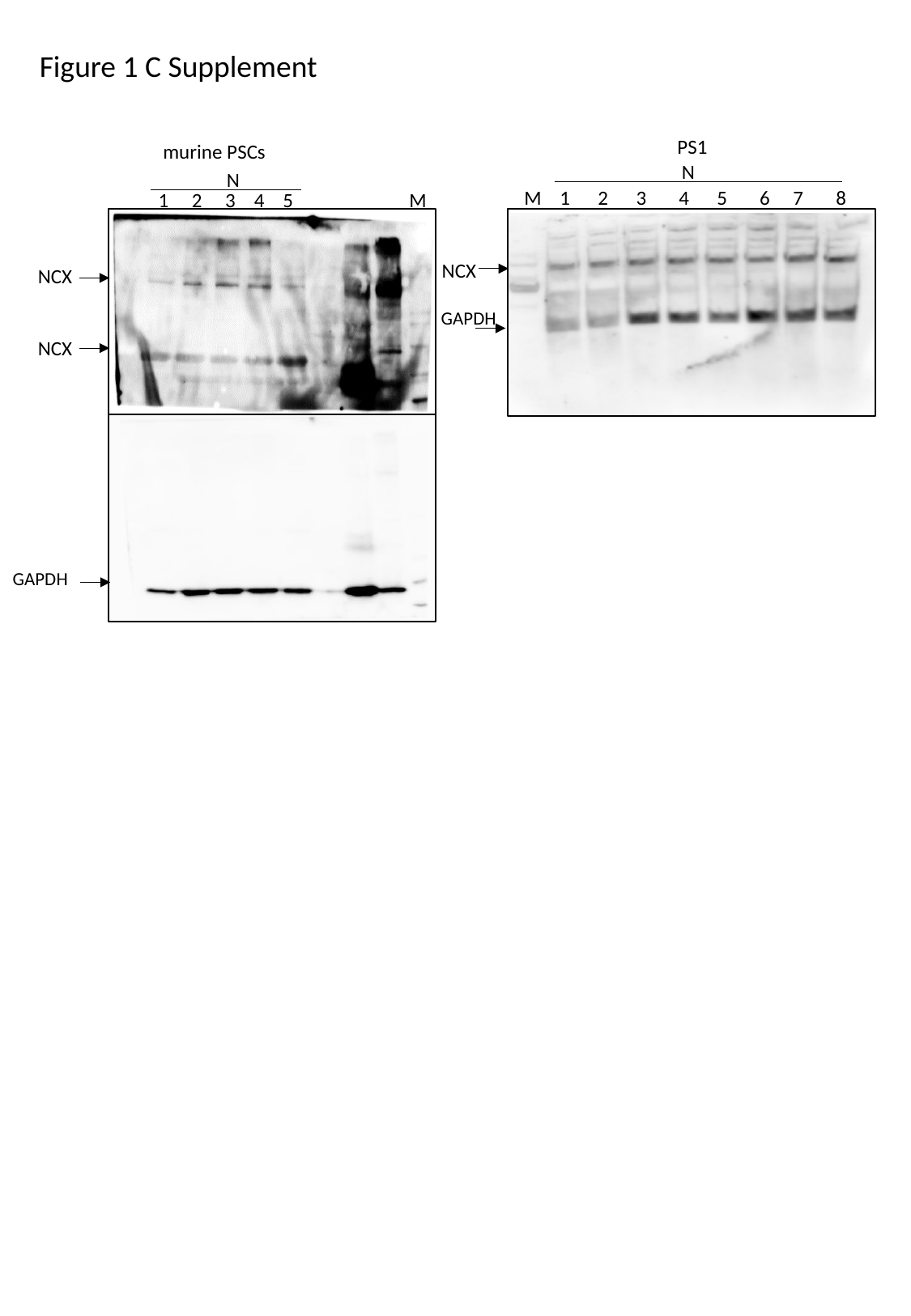

Figure 1 C Supplement
PS1
murine PSCs
N
N
M 1 2 3 4 5 6 7 8
1 2 3 4 5 M
NCX
NCX
GAPDH
NCX
GAPDH

## Slide 2
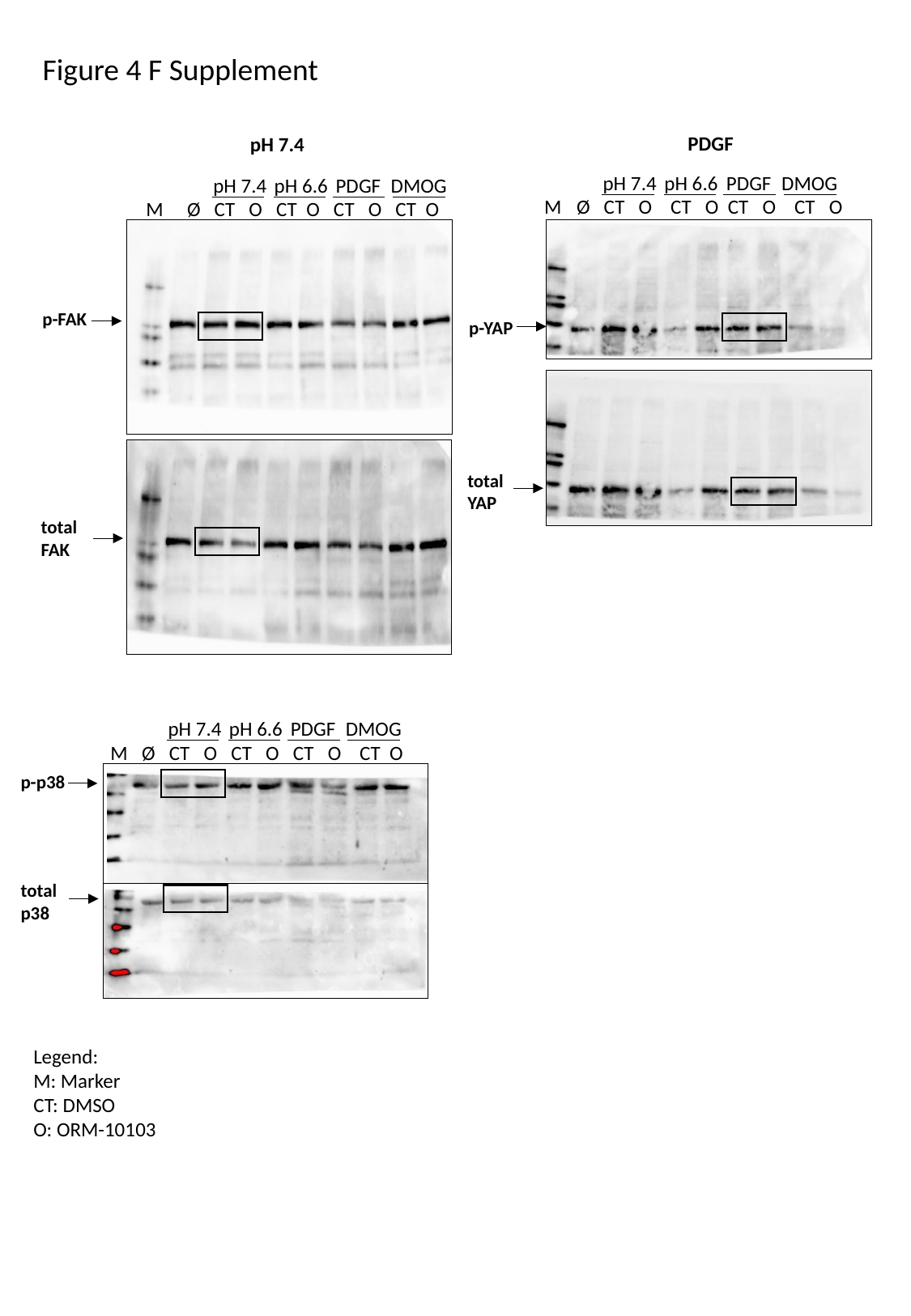

Figure 4 F Supplement
PDGF
pH 7.4
pH 7.4
pH 6.6
PDGF
DMOG
M
Ø CT O CT O CT O CT O
pH 7.4
pH 6.6
PDGF
DMOG
M
Ø CT O CT O CT O CT O
p-FAK
p-YAP
total YAP
total FAK
pH 7.4
pH 6.6
PDGF
DMOG
M
Ø CT O CT O CT O CT O
p-p38
total p38
Legend:
M: Marker
CT: DMSO
O: ORM-10103

## Slide 3
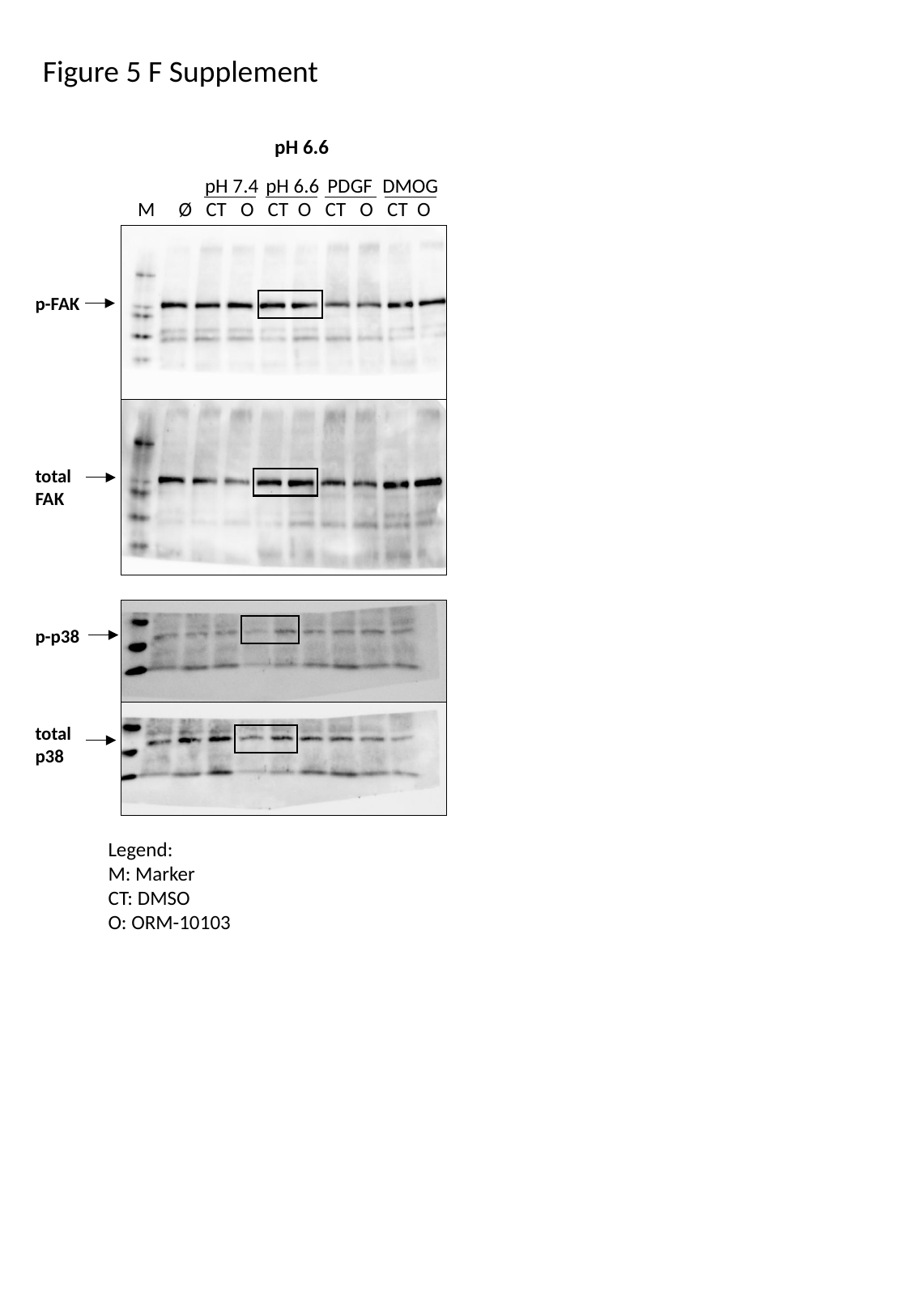

Figure 5 F Supplement
pH 6.6
pH 7.4
pH 6.6
PDGF
DMOG
M
Ø CT O CT O CT O CT O
p-FAK
total FAK
p-p38
total p38
Legend:
M: Marker
CT: DMSO
O: ORM-10103

## Slide 4
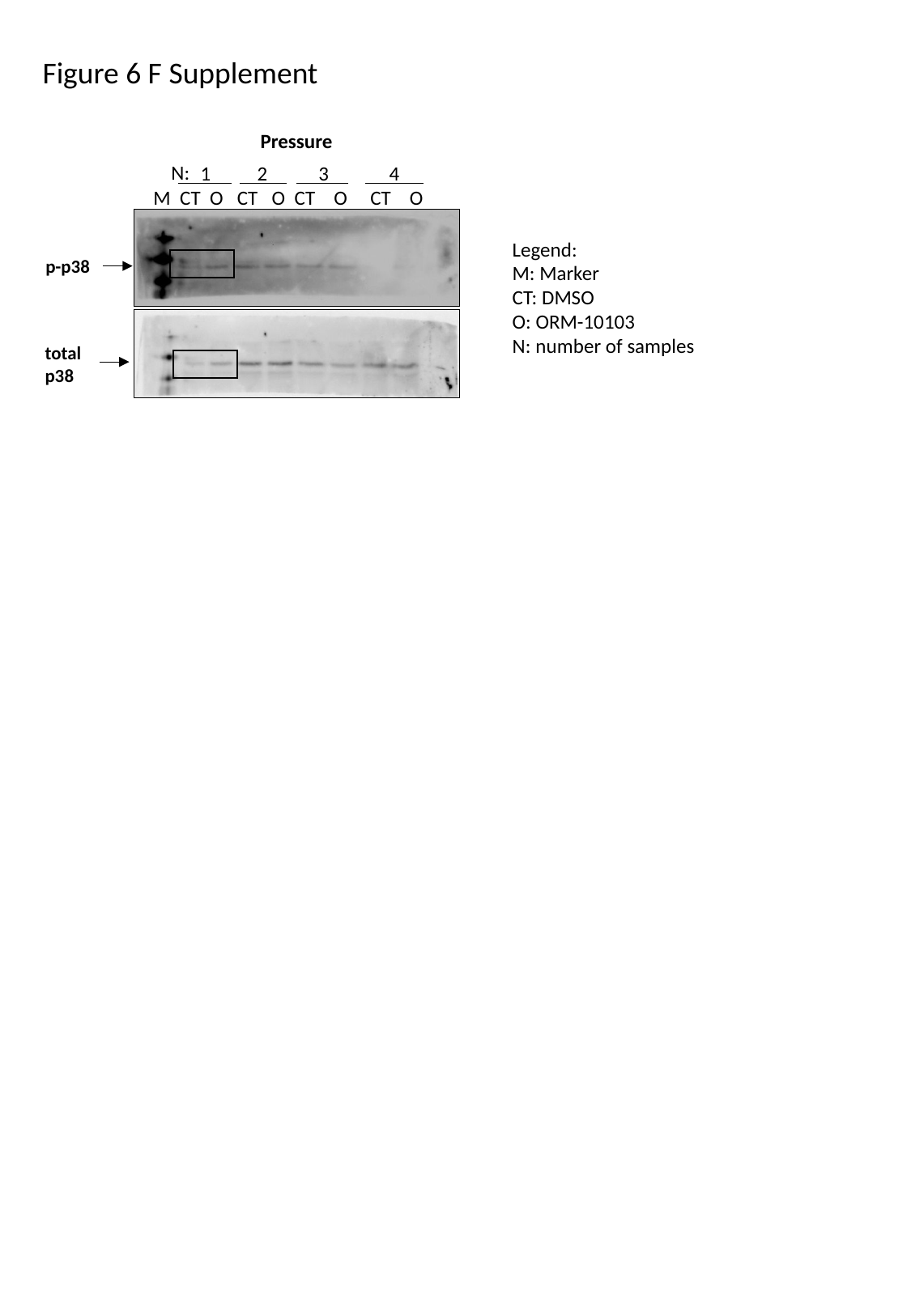

Figure 6 F Supplement
Pressure
N:
1 2 3 4
M CT O CT O CT O CT O
Legend:
M: Marker
CT: DMSO
O: ORM-10103
N: number of samples
p-p38
total p38

## Slide 5
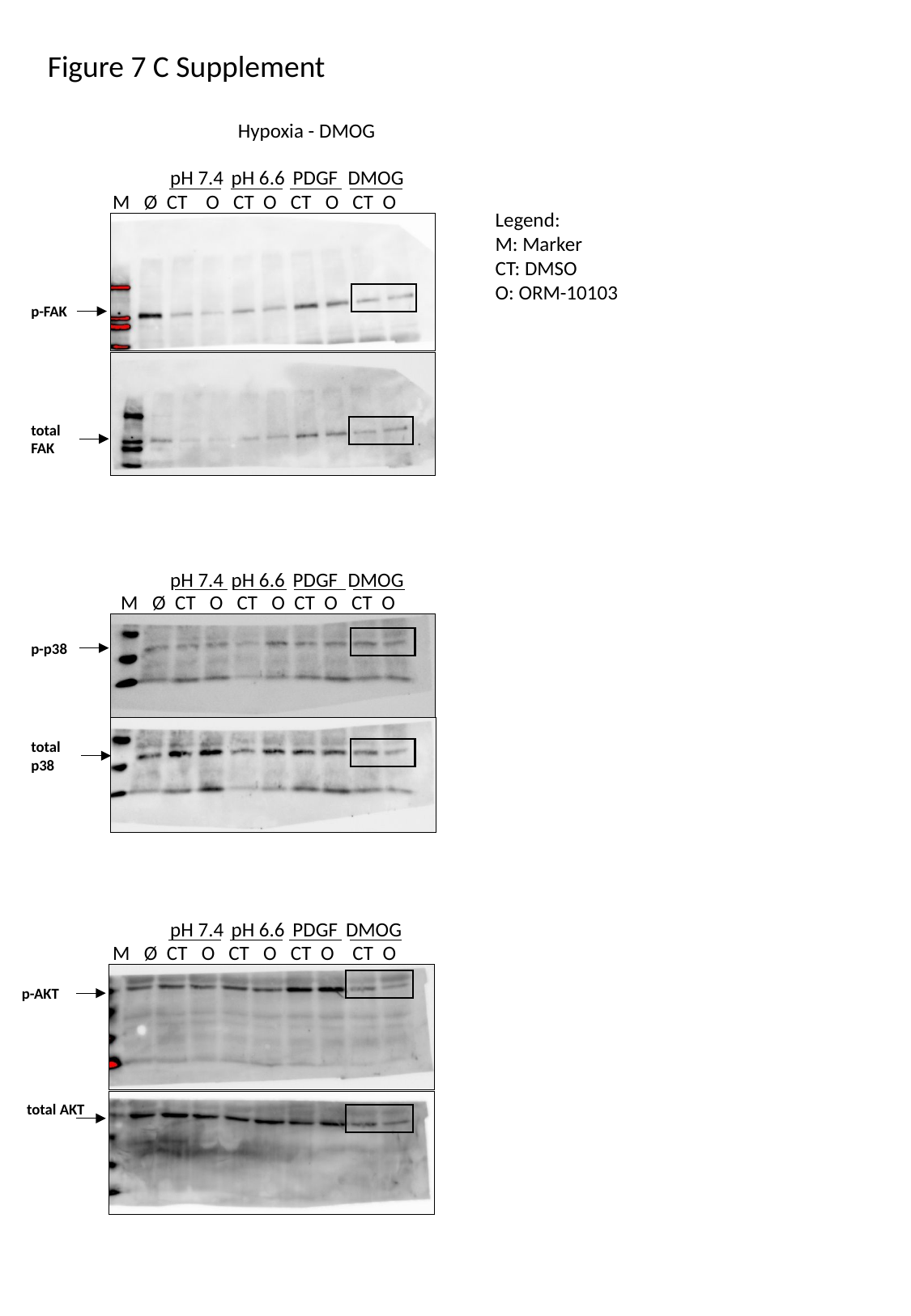

Figure 7 C Supplement
Hypoxia - DMOG
pH 7.4
pH 6.6
PDGF
DMOG
M
Ø CT O CT O CT O CT O
Legend:
M: Marker
CT: DMSO
O: ORM-10103
p-FAK
total FAK
pH 7.4
pH 6.6
PDGF
DMOG
M
Ø CT O CT O CT O CT O
p-p38
total p38
pH 7.4
pH 6.6
PDGF
DMOG
M
Ø CT O CT O CT O CT O
p-AKT
total AKT
